# The *Mutyh* Base Excision Repair Gene Influences the Inflammatory Response in a Mouse Model of Ulcerative Colitis

**DOI:** 10.1371/journal.pone.0012070

**Published:** 2010-08-10

**Authors:** Ida Casorelli, Tania Pannellini, Gabriele De Luca, Paolo Degan, Federica Chiera, Ivano Iavarone, Alessandro Giuliani, Alessia Butera, Monica Boirivant, Piero Musiani, Margherita Bignami

**Affiliations:** 1 Department of Environment and Primary Prevention, Istituto Superiore di Sanità, Rome, Italy; 2 Aging Research Center, Gabriele D'Annunzio University of Chieti, Chieti, Italy; 3 Istituto Tumori di Genova, Genova, Italy; 4 Department of Infectious Parasitic and Immuno-mediated Diseases, Istituto Superiore di Sanità, Rome, Italy; Emory Unviersity, United States of America

## Abstract

**Background:**

The *Mutyh* DNA glycosylase is involved in the repair of oxidized DNA bases. Mutations in the human *MUTYH* gene are responsible for colorectal cancer in familial adenomatous polyposis. Since defective DNA repair genes might contribute to the increased cancer risk associated with inflammatory bowel diseases, we compared the inflammatory response of wild-type and *Mutyh^−/−^* mice to oxidative stress.

**Methodology/Principal Findings:**

The severity of colitis, changes in expression of genes involved in DNA repair and inflammation, DNA 8-oxoguanine levels and microsatellite instability were analysed in colon of mice treated with dextran sulfate sodium (DSS). The *Mutyh^−/−^* phenotpe was associated with a significant accumulation of 8-oxoguanine in colon DNA of treated mice. A single DSS cycle induced severe acute ulcerative colitis in wild-type mice, whereas lesions were modest in *Mutyh^−/−^* mice, and this was associated with moderate variations in the expression of several cytokines. Eight DSS cycles caused chronic colitis in both wild-type and *Mutyh^−/−^* mice. Lymphoid hyperplasia and a significant reduction in Foxp3^+^ regulatory T cells were observed only in *Mutyh^−/−^* mice.

**Conclusions:**

The findings indicate that, in this model of ulcerative colitis, Mutyh plays a major role in maintaining intestinal integrity by affecting the inflammatory response.

## Introduction

Inflammatory processes induce oxidative/nitrosative stress and lipid peroxidation by generating an excess of radical reactive species. Experimental and epidemiological evidences suggested a link between oxidative stress and cancer. Persistent inflammation is considered an important risk factor for colorectal cancer development in Ulcerative colitis (UC) and Crohn's disease, two major Inflammatory Bowel Diseases (IBDs) [1].

Oxidative stress causes different kinds of DNA damage, including single and double strand breaks and base modifications. One of the predominant products, 8-oxoguanine (8-oxoG), is potentially mutagenic and is implicated in carcinogenesis. DNA 8-oxoG codes ambiguously during replication and directs incorporation of C and A with almost equal efficiencies. The mutagenic consequence of 8-oxoG:A mismatches, G∶C->T∶A transversions, are regarded as the signature mutations of DNA 8-oxoG. Base Excision Repair (BER) initiated by the MUTYH DNA glycosylase provides protection against 8-oxoG∶A mispairs [2], [3]. This enzyme removes A incorrectly inserted opposite 8-oxoG. The APE/REF1 endonuclease incises the resulting apurinic site and insertion of a C across the 8-oxoG creates a substrate for enzymes which remove this oxidized purine [4]. In addition, Mutyh can remove other sources of potentially mutagenic lesions such as oxidized adenines [5]. The absence of this repair pathway in *Mutyh* ^−/−^ cells is associated with accumulation of DNA 8-oxoG and a mutator phenotype [6], [7]. In addition, *Mutyh*-null mice show enhanced susceptibility to spontaneous and KBrO_3_-induced intestinal carcinogenesis [8] and Mutyh deficiency enhances intestinal tumorigenesis in *Apc^min/+^* mice [9]. Biallelic germ-line mutations in human *MUT*YH have been identified in patients affected by colorectal adenomatous polyposis [10]. Accumulation of somatic GC->TA transversions in *APC* and *KRAS*, most likely because of defective repair of 8-oxoG∶A mismatches, characterize this autosomal recessive syndrome [11], [12].

Oxidative stress in IBDs is associated with increased levels of oxidized DNA bases [1]. Furthermore the accumulation of 8-oxoG in the mucosa of UC-associated neoplasia accompanied by functional inactivation human MUTYH have been proposed as early events in UC-associated carcinogenesis [13]. Since DNA repair genes might play a role in cancer risk associated with chronic inflammation occurring in IBDs [1], [14], in this study we compared the response of *Mutyh* ^−/−^ and wild-type (wt) mice to single or multiple cycles of dextran sulfate sodium (DSS) in drinking water. DSS induces colonic oxidative stress and colitis that mimics human IBDs [15]. In the acute phase, exposure of the mucosa to bacterial antigens generates an acute inflammatory response and damage to the epithelium. In the chronic phase, the mucosa is still inflamed although the epithelium is restored, albeit with an irregular structure. Long-term DSS exposure can be associated with the induction of colonic tumors. Indeed DSS has been shown to enhance 8-oxoG levels in rat and mouse colonic mucosa [16], [17].

Following a DSS-induced oxidative stress we investigated the role of Mutyh in modulating levels of DNA oxidation and patterns of expression of 40 genes involved in the inflammatory response or in DNA repair. Our findings indicate that the inflammatory response elicited by acute DSS exposure is diminished in *Mutyh^−/−^*mice in comparison to their wt counterparts. In contrast, a chronic exposure to DSS induces an inflammatory response in both wt and *Mutyh^−/−^* mice, although a more florid lymphoid hyperplasia with a significant reduction of Foxp3^+^ regulatory T cells (Tregs) is apparent in the latter animals.

## Results

### Acute colitis

**Figure 1:**
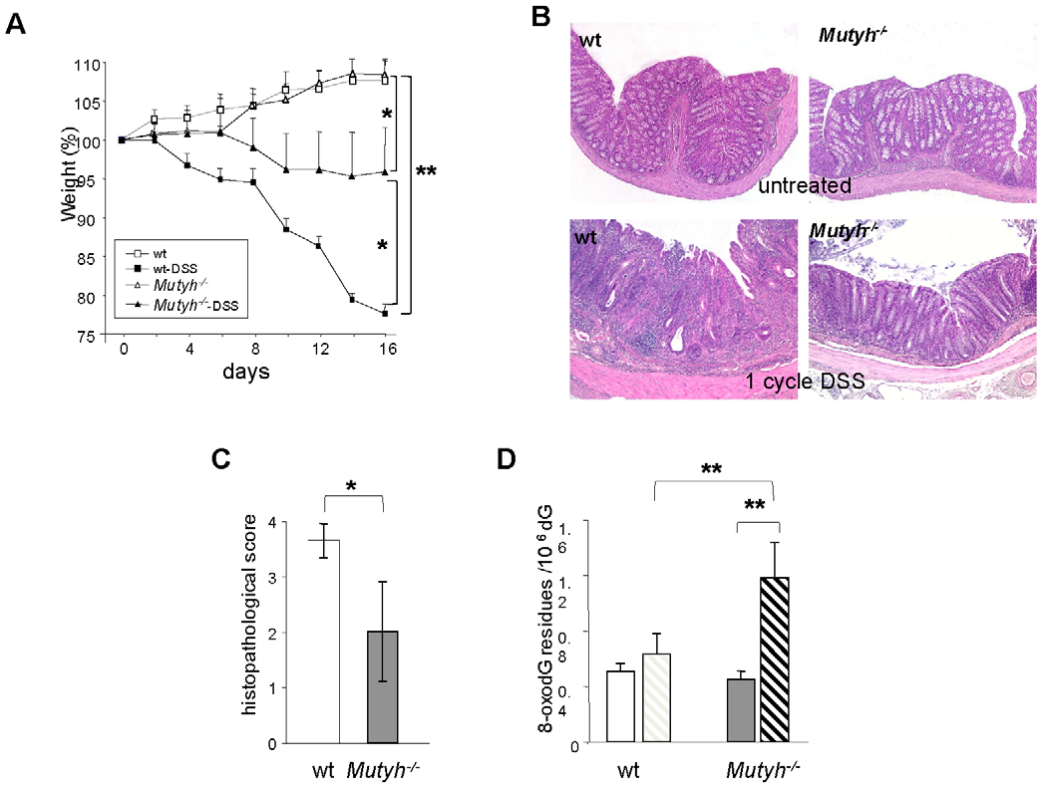
Acute colitis induced by one DSS cycle. Panel A. Effects on body weights following 3% DSS administration in 10 wt and 7 *Mutyh^−/−^* mice. Body weight variations are shown as percentage of weights at the beginning of the analysis in the control or DSS-treated groups. Data are mean ± SE. *P* values were calculated by comparing wt and *Mutyh^−/−^* mice at the same days of DSS treatment. Untreated wt (open circles), DSS-treated wt (closed circles); untreated *Mutyh^−/−^* (open triangles), DSS-treated *Mutyh^−/−^* (closed triangles). Panel B. Photomicrographs of colon from wt (left) and *Mutyh−/−* (right) mice exposed (lower row) or not (upper row) to one DSS cycle. Severe colitis in wt animals is indicated by ulcerations of the mucosa, crypt erosion and abscesses, goblet cell reduction, oedema and marked presence of inflammatory cells in the lamina propria. A reduction in the number of epithelial lesions and infiltrating inflammatory cells is observed in Mutyh−/− mice. Lesions correspond to an average histopathological score of 3 and 1 in wt and Mutyh−/− mice, respectively. Scale bars: 100 mm. Panel C. The extent of inflammation was calculated at the end of treatment as histopathological score according to indicated criteria (Table 1 and ref. [15]). Data are mean + SE of 11 wt and 6 *Mutyh−/−* mice. Panel D. Levels of DNA 8-oxoG in colonic mucosa of untreated and DSS-treated mice. Data are the mean + SE from 20 untreated (open bars) and 10 DSS-treated wt (dashed bars) and 8 untreated (grey bars) and 5 DSS-treated *Mutyh−/−* mice (dashed bars). DSS-treated groups are compared to untreated controls by t-tests and two-way P-values (*P = 0.05; ** P<0.05).

A single cycle of 3% DSS for 7 days followed by a 10-day period with water (1 cycle) induced acute colitis. In wt animals, colitis was associated with decrease of body weight in comparison to controls (*P*<0.01 from day 4 of DSS exposure)(Figure 1A). Weight loss was less pronounced in *Mutyh^−/−^* animals (*P*<0.05, days 14^th^ and 16^th^) and their weights differed significantly from untreated mice only at the end of the DSS treatment (*P*<0.05, day 16)(Figure 1A).

Histopathological analyses of colonic sections from wt mice, revealed signs of frank inflammation and confirmed the presence of severe acute UC in most animals (10/11, >90%). Colitis was characterized by the presence of mucosal ulcerations, infiltration of the lamina propria by inflammatory cells, oedema, crypt erosion and abscesses, gland loss, goblet cell reduction and signs of epithelial regeneration (Figure 1B). These features were significantly attenuated in *Mutyh^−/−^* mice and only 50% showed signs of inflammation. Lesions were either absent or modest and we observed few ulcers (Figure 1B). Table 1 details the severity of colitis quantified from histological scores [15], with minor modifications. The mean histopathological scores for colitis in wt animals were significantly higher than those of *Mutyh^−/−^* mice (*P* = 0.05)(Figure 1C).

**Table 1:** Scoring system for inflammation-associated histological changes in the colon of DSS -treated mice.

Colitis Grade	Histological Parameters
0	No evidence of inflammation
1	Slight colitis: low level of inflammation with scattered infiltrating granulocytes, monocytes and plasmacells (1–2 foci).
2	Moderate colitis: moderate inflammation with multiple foci, oedema of lamina propria, scattered granulocytes.
3	Severe colitis: high level of inflammation with marked wall thickening, oedema of the lamina propria, glands distortion, gland erosion and abscesses, ulcerations of superficial epithelial cells.
4	Maximal severity of inflammation with transmural leukocyte infiltration, almost complete loss of goblet cells, large ulcerations involving all mucosal wall and deep suppuration foci.

Since DSS is known to induce oxidative stress we investigated whether this treatment resulted in the accumulation of 8-oxoG in colon DNA (Figure 1D). Acute DSS treatment increased the amount of DNA 8-oxoG in *Mutyh^−/−^* mice >2.5-fold compared to untreated controls. The levels of this oxidized base remained unchanged in wt mice (Figure 1D). As previously reported [18], basal levels of 8-oxoG in colon DNA were unaffected by Mutyh inactivation.

These findings indicate that the inflammatory response to DSS exposure in *Mutyh^−/−^* mice is attenuated in comparison to that in wt animals. This is accompanied by the accumulation of significantly higher levels of DSS-induced DNA 8-oxoG in the colon of *Mutyh^−/−^* animals.

### Gene expression in DSS–induced acute colitis

**Figure 2:**
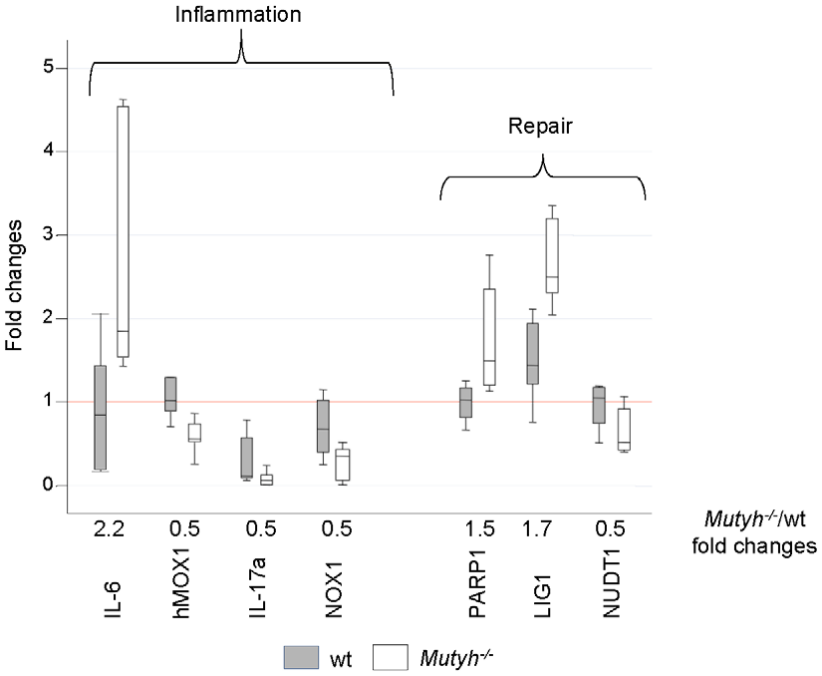
Expression levels of selected genes in untreated wt and *Mutyh−/−* mice. Box Plots of fold-changes in the expression of genes are shown for untreated wt and *Mutyh−/−* mice (8 and 7 animals, respectively). For each gene, fold-change ratios between *Mutyh−/−* and wt groups are also displayed. A representative untreated wt colon sample was chosen as calibrator. Only genes with statistically significant variations in the expression levels are shown. wt (closed symbol), *Mutyh−/−* mice (open symbols).

Gene expression in colon samples from 20 wt and 19 *Mutyh^−/−^* animals was analysed using low-density arrays containing 40 genes involved in DNA repair and inflammatory responses (Table 2). Samples were classified on the basis of genotypes and treatment. In untreated animals, only a few genes differed in basal expression between the two genotypes. In *Mutyh^−/−^* mice, expression of hMOX1, IL-17A and NOX1 was 2-fold lower (*P*<0.05) and IL-6 (*P* = 0.02) was 2-fold higher than in wt mice (Figure 2). Significant differences in steady-state levels of DNA repair gene mRNA were also observed. LIG1 and PARP1 levels were higher and NUDT1 expression was lower in *Mutyh^−/−^* mice (*P*≤0.05) (Figure 2).

**Table 2:** List of genes involved in the inflammatory response and in DNA repair and analyzed for expression.

CODE	GENE	CODE	GENE
*INFLAMMATORY RESPONSE*		*RECOMBINATIONAL REPAIR*	
Mm00443258_m1	*TNF*α	Mm00450600_m1	*Mre11a*
Mm00484741_m1	*SMAD7*	Mm00487905_m1	*RAD51*
Mm00489637_m1	*SMAD3*	Mm00550147_m1	*XRCC5 (KU70)*
Mm00478374_m1	*PTGS2*	Mm00487458_m1	*XRCC6 (KU80)*
Mm00477214_m1	*PTGS1*	Mm00465092_m1	*PRKDC (DNA-PK)*
Mm00500821_m1	*NQO1*		
Mm00549170_m1	*NOX1*	*MISMATCH REPAIR*	
Mm00440485_m1	*NOS2*	Mm00500563_m1	*MSH2*
Mm00479807_m1	*NFKB2*	Mm00487756_m1	*MSH3*
Mm00446190_m1	*IL6*	Mm00487761_m1	*MSH6*
Mm00518984_m1	*IL23a*	Mm00503449_m1	*MLH1*
Mm00434228_m1	*IL1b*	Mm00476986_m1	*PMS2*
Mm00444686_m1	*IL17b*		
Mm00439619_m1	*IL17a*	*BASE EXCISION REPAIR*	
Mm00434204_m1	*IL13*	Mm00521933_m1	*LIG3*
Mm00434165_m1	*IL12a*	Mm00495331_m1	*LIG1*
Mm00439616_m1	*IL10*	Mm00494229_m1	*XRCC1*
Mm00801778_m1	*IFN*Γ	Mm00448234_m1	*POLB*
Mm00516004_m1	*HMOX1*	Mm00507805_g1	*APEX1*
Mm00599683_m1	*CD3E*	Mm00449156_m1	*UNG*
		Mm00500154_m1	*PARP1*
		Mm00447872_m1	*MPG*
DNA DAMAGE RESPONSE		DAMAGE AVOIDANCE	
Mm00458141_m1	*TRP53inp1*	Mm00599710_m1	*NUDT1*

**Figure 3:**
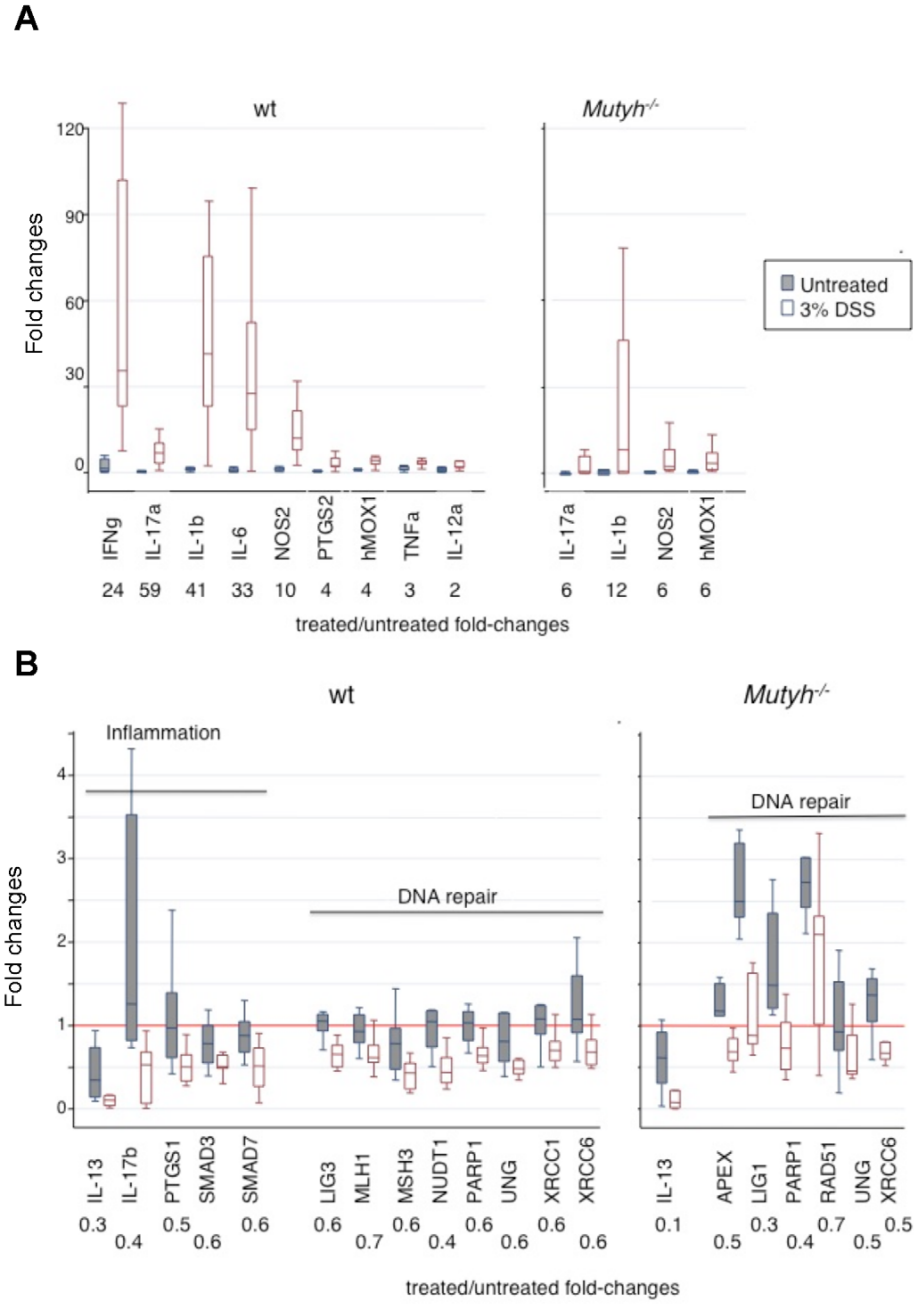
Changes in gene expression induced by one DSS cycle. Box Plots of fold-changes in the expression of up-regulated (Panel A) or down-regulated genes (Panel B) for untreated and DSS-treated wt (left) (8 and 12 animals, respectively) and *Mutyh−/−* mice (right)(7 and 12 animals, respectively). For each gene fold-change ratios between treated and untreated groups are also displayed. A representative untreated wt colon sample was chosen as calibrator. Only genes with statistically significant variations in the expression levels are shown.

Acute DSS treatment induced widespread changes in gene expression in the colon of wt mice (Figure 3A–B). Acute colitis was associated with up-regulation of several genes involved in pro-inflammatory pathways (IFNγ, IL-17a, IL-1b, IL-6, NOS2, PTGS2, hMOX1, TNFα and IL-12a) (*P*<0.01). hMOX1 was induced to a similar degree in *Mutyh^−/−^* mice but changes in IL-17a, IL-1b and NOS2 were less pronounced (*P*≤0.01) (Figure 3A) and there was no significant increase in expression of IL-12a, IL-6, PTGS2, or TNFα (data not shown). Strikingly, there was no up-regulation of IFNγ in *Mutyh^−/−^* mice.

Another set of genes, the majority of which encode DNA repair factors, was significantly down-regulated by acute DSS treatment (Figure 3B). The extent of these changes was less marked (2-fold). In *Mutyh^−/−^* mice the down-regulation was restricted to a subset of these genes, although the degree of variation was similar in the two genotypes. The only down-regulated gene of the inflammatory panel, IL-13, was affected more significantly in *Mutyh^−/−^* than in wt mice (10- versus 3-fold change).

In conclusion following an acute DSS treatment, *Mutyh^−/−^* mice display a lower number of modulated genes as compared to wt animals, when either up-regulated (4 *versus* 9 genes) or down-regulated genes (7 *versus* 13) are taken into consideration. Overall, the limited modulation of pro- or anti-inflammatory cytokines in *Mutyh^−/−^* mice nicely parallels the moderate degree of inflammation observed histologically in these animals.

### Chronic colitis

**Figure 4:**
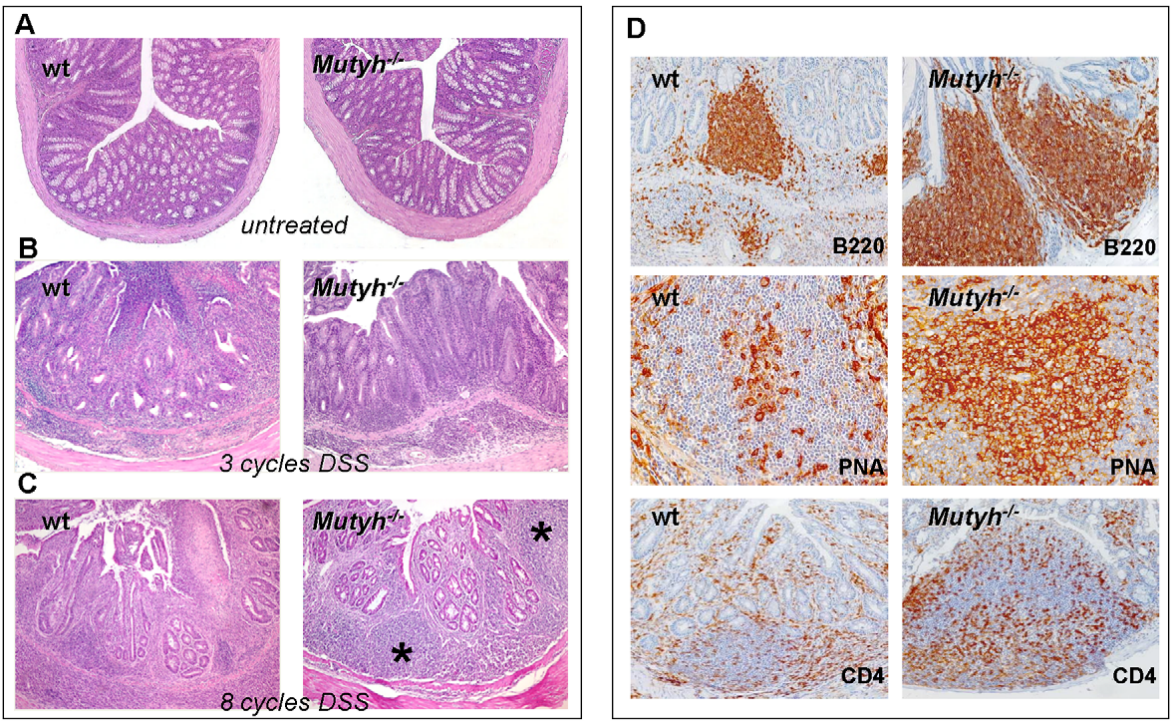
Histological analysis of colon from untreated or DSS-treated wt and *Mutyh−/−* mice. After three DSS cycles (Panel B), wt mice show a severe acute UC with several lesions of the glandular epithelium, ulcers and a marked increase of reactive cells. The colitis in *Mutyh−/−* mice is less aggressive with few ulcers and a moderate increase of reactive cells (100× magnification). After eight DSS cycles (Panel C), chronic UC in *Mutyh−/−* is less severe than in wt mice. In *Mutyh−/−* mice numerous and ample lymphoid follicles (asterisks) are present in the lamina propria or between this and submucosa (100× magnification). After eight DSS cycles (Panel D), lymphoid hyperplasia occurring in Mutyh−/− mice is formed by B220+ cells, PNA+ GCs filled with centroblasts and centrocytes and CD4+ T lymphocytes, mostly located around follicles and in the interfollicular zones. In wt animals the number of B220+ cells, PNA+ GCs and CD4+ T lymphocyte is lower than in Mutyh−/− (B220 and CD4: 200× magnification; PNA: 400× magnification).

After three 1.5% DSS cycles, severe lesions of the glandular epithelium and ulcers coupled with a decrease in the number of mucus-secreting cells and a marked increase in the number of reactive cells in the lamina propria and submucosa similar to those of acute UC were observed in wt mice (mean histopathological score 3.7±0.5 and Figure 4B). In contrast, colitis in *Mutyh^−/−^* mice was less aggressive with few ulcers, the lesions of the glandular epithelium were focally distributed and the lamina propria and submucosa were moderately infiltrated by reactive cells (mean histopathological score 2.7±0.4 and Figure 4B).

Eight DSS courses resulted in chronic colitis in wt mice with residual glands coated with an epithelium containing few mucus-secreting cells (Figure 4C). Numerous reactive cells with lymphoid cells, sometimes arranged in follicles, occupied the lamina propria and the submucosa. Often granulation tissue filled in the ulcer craters, followed by regeneration of the mucosal epithelium. In some areas submucosal fibrosis and mucosal architectural disarray and atrophy remained as residual of healed disease. The same treatment induced chronic UC in *Mutyh^−/−^* mice, although this was less severe. *Mutyh^−/−^* mice had numerous ample lymphoid follicles at the level of the colonic lamina propria or between this and the submucosa. A hyperplastic mantle zone and florid Germinal Centers (GCs) characterized these follicles, with a large number of leukocytes located in inter-follicular zones (Figure 4C). Neither wt nor *Mutyh^−/−^* mice showed neoplastic transformation of mucosal glands.

Immunohistochemical analysis of lymphoid aggregates revealed an impressive lymphoid hyperplasia of B220+ B lymphocytes in *Mutyh^−/−^* mice (Figure 4D). These were organized in PNA+ GCs and were more pronounced and larger than in wt mice. Numerous CD4+ T lymphocytes were present in the inter-follicular zones and at the borders of the follicles, again in larger numbers in *Mutyh^−/−^* mice (Figure 4D), although the architecture of these lymphoid structures did not show gross deviations from the normal structure.

**Figure 5:**
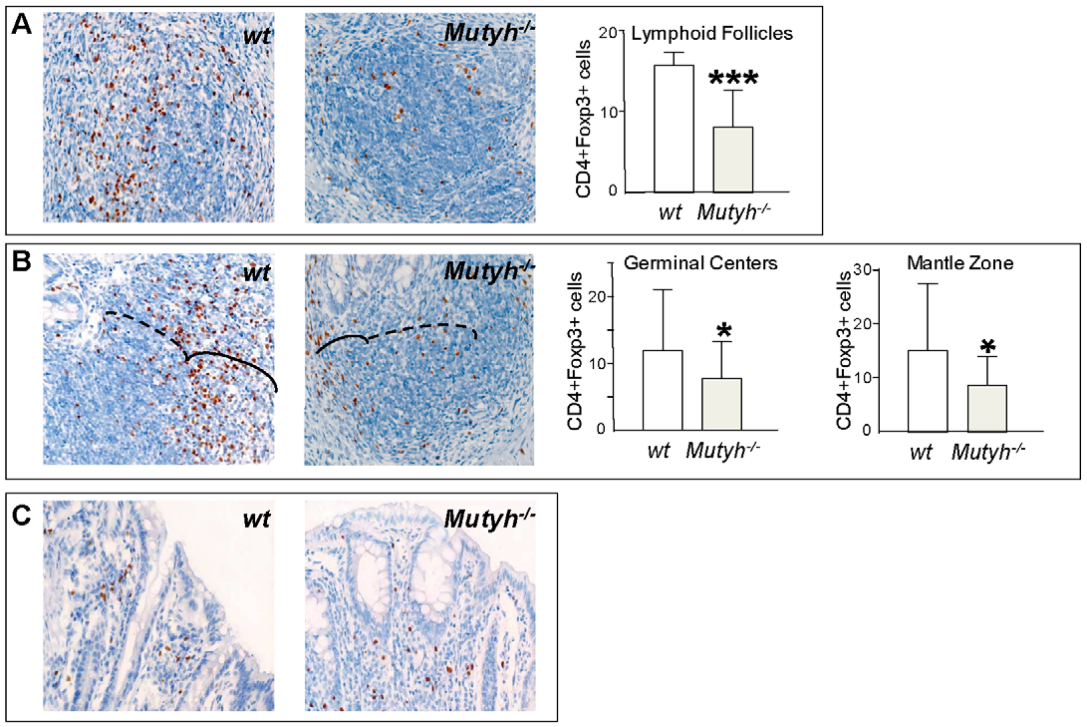
CD4+ Foxp3^+^ cells quantisation in colon samples from mice exposed to eight DSS cycles. Following eight DSS cycles, *Mutyh^−/−^* mice animals show a reduction in the number of Foxp3^+^ cells in lymphoid follicles without active GCs (A), in GCs (solid line in B) or in the Mantle Zone (dotted line in B) in comparison to wt mice. No differences were found in the Foxp3^+^ cells scattered in the lamina propria (C). The number of CD4+ Foxp3^+^ cells is reported as the number of CD4+ Foxp3^+^ cells/number of Mantle Zone, or GCs, or Lymphoid Follicles (400× magnification). Unpaired t test. * p< 0.05, *** p< 0.005.

In comparison to wt mice, the lymphoid hyperplasia in *Mutyh^−/−^* mice was always associated with very low levels of CD4+/Foxp3^+^ T regs (Figure 5A–B). These cells were significantly diminished both in follicles without (Figure 5A) and with GCs (continuous line, Figure 5B) as well as in the mantle zone (dotted line, Figure 5B). No difference was observed between the two genotypes in the relatively low number of Foxp3^+^ cells scattered in the lamina propria and intraepithelia (Figure 5C).

In summary, the extent of inflammation associated with chronic DSS exposure differs quantitatively and qualitatively between wt and *Mutyh^−/−^* mice. In particular, the marked lymphoid hyperplasia in *Mutyh^−/−^* mice is associated with very low levels of CD4+/Foxp3^+^ Tregs.

### Gene expression in chronic colitis

**Figure 6:**
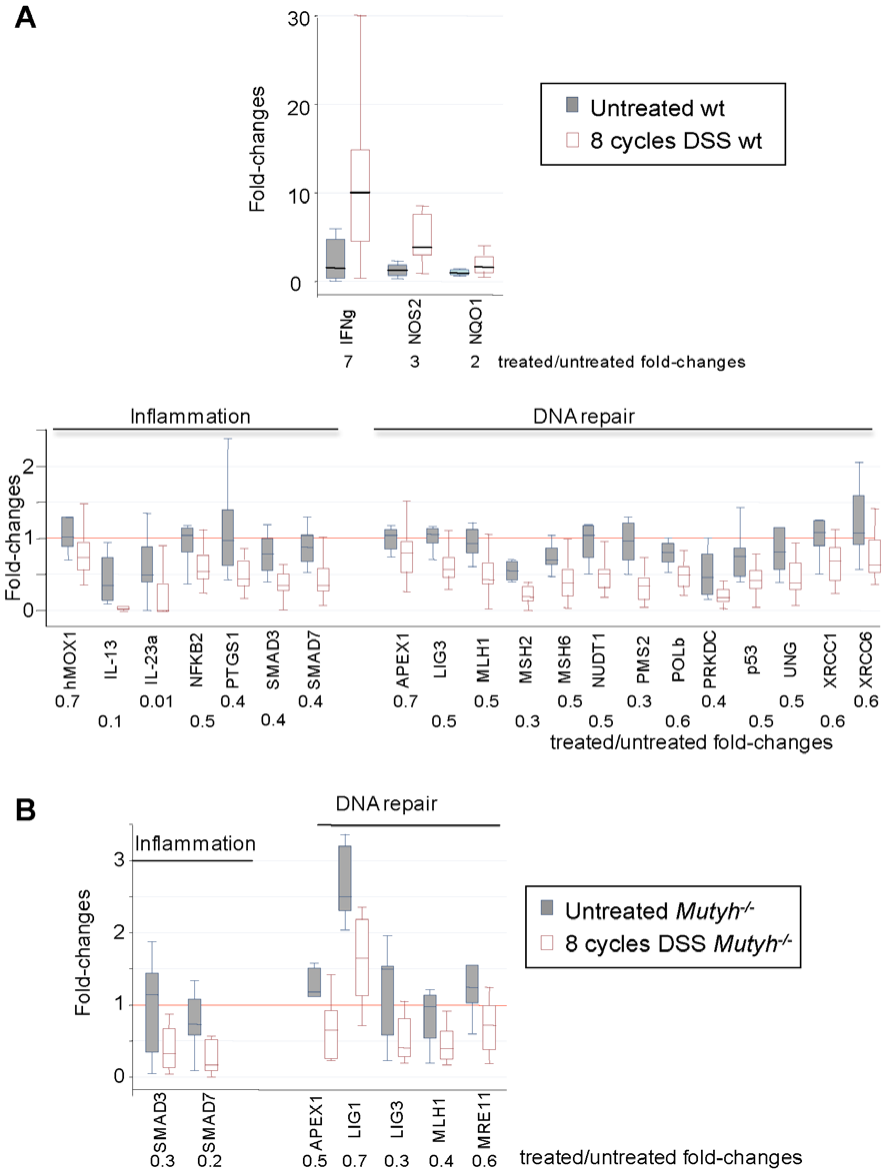
Changes in gene expression induced by eight DSS cycles. Panel A. Box Plots of fold-changes in the expression of up-regulated (top) and down-regulated (bottom) genes are shown for 8 untreated and 16 DSS-treated wt mice. Panel B. Box Plots of fold-changes in the expression of down-regulated genes are shown for 7 untreated and 8 DSS-treated *Mutyh^−/−^* mice.

The progression from acute to chronic colitis was associated with more limited changes in inflammatory response genes. In wt mice there were fewer genes with significantly altered expression (3 *versus* 9). Only IFNγ, NOS2 and NQO1 (*P*≤0.01) were up-regulated after chronic DSS treatment (Figure 6A). The extent of induction was also lower compared to acute colitis. Examples include IFNγ (7- *versus* 24-fold) and NOS2 (3- *versus* 10-fold)(Figure 3A and Figure 6A). None of these genes showed a statistically significant change in *Mutyh^−/−^* mice, although a trend towards up-regulation of IFNγ, IL-12a, IL-6 was observed (data not shown).

In wt mice with chronic colitis, IL-23a, IL-13, NF-kB2, PTGS1, SMAD3 and SMAD7 (*P*≤0.05) were significantly down-regulated, with the largest variations affecting the expression of IL-23a and IL-13 (100- and 10-fold) (Figure 6A).

A smaller subset of genes was down-regulated in the colon of *Mutyh^−/−^* mice (7 *versus* 20 in wt mice), and these included SMAD3, SMAD7, APEX1, LIG3, MLH1, LIG1 and MRE11 (*P*≤0.05) (Figure 6B).

Overall, the differences in inflammation between acute and chronic colitis are paralleled by differences in the expression of pro-inflammatory cytokines. In contrast the down-regulation of DNA repair genes is largely unaffected by the shift from acute to chronic colitis. The response of *Mutyh^−/−^* mice to a chronic DSS exposure is more restricted with altered expression of fewer genes in both categories.

Gene expression data were also analyzed by another statistical method, Principal Component Analysis (PCA). This unsupervised learning approach investigates natural co-expression structures (components) of the analysed genes. A significant *a posteriori* discrimination between samples from treated and untreated mice was accomplished by the first five components as shown by the analysis of variance on the component scores (Figure S1). The global concordance of analyses gene-by-gene and by a multi-dimensional perspective is a robust proof-of-concept of the existence of strict links between DNA repair and the inflammatory response in terms of gene expression.

### DNA oxidation and microsatellite instability

**Figure 7:**
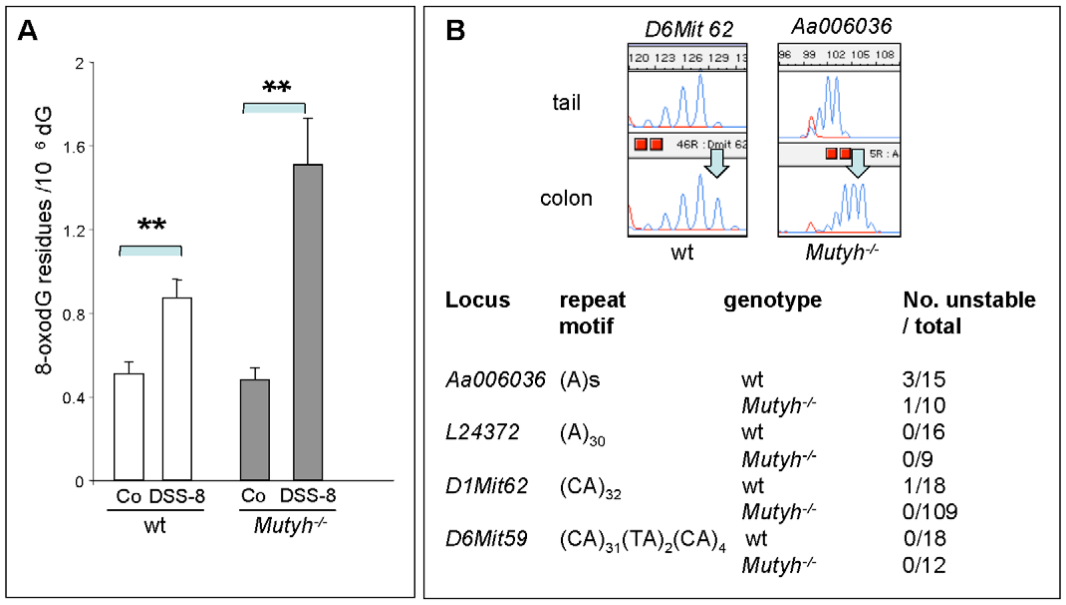
DNA 8-oxoG and MSI levels in colonic mucosa of mice exposed to eight DSS cycles. Panel A. Data are the mean ± SE of measurements from wt (open bars) and *Mutyh^−/−^* mice (closed bars) (20 animals per genotype) ** P<0.05.Panel B. The numbers of mice with MSI are shown together with examples of unstable *D6Mit62* and *Aa006036* loci. The arrows indicate the shift in the highest peak.

The levels of DNA 8-oxoG in the colon of wt mice increased 1.7-fold after chronic DSS treatment (Figure 7A). The increased accumulation in the DNA of *Mutyh^−/−^* mice was 2-fold higher (Figure 7A).

MSI in colon DNA was investigated using four microsatellite probes (*Aa006036, L24372, D1Mit62, D6Mit59*) (Figure 7B). A very limited MSI was identified in DSS-treated animals at two loci (*Aa006036* and *D1Mit62*). There were no apparent difference between wt and *Mutyh^−/−^* mice in this regard.

These findings indicate that chronic DSS treatment results in a more pronounced accumulation of 8-oxoG in colon DNA of *Mutyh^−/−^* mice than in their wt counterparts. This increased amount of oxidized DNA bases is not accompanied by widespread MSI in both genetic backgrounds.

## Discussion

In this study of the effect of Mutyh deficiency on the inflammatory response, we used the DSS mouse model for IBDs. In view of the known substrate specificity of Mutyh [3] and the association between DSS treatment and increased levels of DNA 8-oxoG [16], [17], we measured this lesion in colon DNA of wt and *Mutyh^−/−^* mice. Several cycles of DSS were required to significantly increase 8-oxoG levels in wt animals whereas the accumulation of the lesion was evident in *Mutyh^−/−^* mice after a single DSS cycle and the levels increased further with chronic exposures. These results indicate that Mutyh is an important barrier against accumulation of DNA damage during oxidative stress associated with UC. High levels of oxidative DNA damage have also been reported in UC-inflamed human colonic mucosa [19] accompanied by altered expression of MUTYH [13].

Accumulation of DNA 8-oxoG was not accompanied by high levels of MSI in either wt or Mutyh-defective mice, even after prolonged periods (6 months). These findings are in agreement with reports that chronic oxidative stress being associated with low-MSI [1].

DSS treatment caused down-regulation of DNA repair genes involved in different DNA repair pathways. We did not observe any DNA repair gene up-regulation. This contrasts to the up-regulation of some BER genes (*APE1, AAG*) in human IBDs [14]. Whether this is due to differences between the mouse model and human IBDs remains to be clarified.

The most striking observation of our study is the significant and unexpected role of Mutyh in modulating the inflammatory response occurring in DSS-induced colitis [20]. Histopathological examination and gene expression analyses concur that *Mutyh^−/−^* mice are less prone to the induction of acute colitis by DSS. Mutyh modulates DSS-mediated inflammation both quantitatively and qualitatively. Thus, the reduced inflammation in *Mutyh^−/−^* mice is reflected in fewer changes in gene expression as well as in reduced induction of the pro-inflammatory cytokines IL-17a, IL-1b, IL-6 and no variation in IFNγ. The expression of inducible nitric oxide synthase NOS*2* - an important mediator of intestinal inflammation in this colitis model [21] - is also reduced. The qualitative difference in the inflammatory response is exemplified by the prevalence of Th17 *vs* Th-1/Th-17 response [22] and suggests that Mutyh might act as an inflammatory/immune system modulator affecting DSS-mediated inflammation both quantitatively and qualitatively. The anti-inflammatory IL-13 cytokine was significantly down-regulated in both wt and *Mutyh^−/−^* mice. This indicates that Mutyh deficiency, while contributing significantly to the induction of the pro-inflammatory response to DSS, does not significantly affect anti-inflammatory mechanisms.

Chronic inflammatory stimuli underlined the involvement of Mutyh in the inflammatory response. Chronic colitis in *Mutyh^−/−^* mice was accompanied by an obvious lymphoid hyperplasia, with lymphocytes organized in follicles often characterized by the presence of large GCs. Similarly to previous reports [23], progression from acute to chronic colitis in wt mice was associated with a reduced expression of IFNγ and IL-6, accompanied by an increased (albeit not significant) IL-10 levels. In contrast chronically-treated M*utyh^−/−^* mice show a trend towards up-regulation of IFNγ, IL12a and IL-6 suggesting that the chronic inflammation occurring in these mice is characterized by the persistence of cytokines dominating the acute phase. Taken together these data suggest that Mutyh somehow contributes to the cellular adaptation towards chronic inflammatory stimuli.

This view is supported by the striking decrease in CD4+/Foxp3^+^ Tregs in chronically treated *Mutyh^−/−^* mice. Tregs control the bacteria-driven inflammatory response in the gastrointestinal tract [24], [25]. In experimental models of colitis, the appearance of increased numbers of Foxp3^+^ cells precedes the spontaneous resolution of inflammation, whereas a low number is associated with persistence of inflammation [26]. Thus intense lymphoid hyperplasia observed in the colon of in *Mutyh^−/−^* mice is consistent with the observed reduction in the number of Tregs. Of interest, the reduction of CD4+/Foxp3^+^ cells in the follicles might be the conversion of Foxp3^+^ T cells into follicular (CD4+/Foxp3^−^) helper T cells, as recently described in mouse Peyer's patches [27]. These follicular CD4+/Foxp3^−^ helper T cells might cooperate with B cells in inducing the formation of GCs in colon mucosa and contribute to establishing and chronically-maintaining the observed lymphoid hyperplasia.

A limited set of genes was also affected by Mutyh inactivation in untreated animals. The decreased IL-17a level together with an increased IL-6 observed in *Mutyh^−/−^* in comparison to wt animals suggest that Mutyh germline inactivation might modify both the immune response to external stimuli and baseline protective immunity.

The limited inflammatory response induced by various inflammatory stimuli (LPS-induced endotoxic shock, streptozotocin-induced Type I diabetes, oxazolone-induced contact hypersensitivity and *Helicobacter pylori*-induced gastritis) in mice in which Ogg1, the DNA glycosylase involved in the removal of 8-oxoG [28], is inactive is also consistent with the involvement of oxidized DNA guanine in inflammation [29], [30]. In addition long exposures to DSS (15 cycles) significantly increased adenocarcinoma development in the colon of *Ogg1^−/−^* in comparison to wt mice, even in the absence of carcinogen treatment [31]. This appears to be a specific response to oxidative DNA damage and other DNA repair-defective animals (*Aag^−/−^*, *Msh2^−/−^* and *Mlh1^−/−^* mice) are more susceptible to colorectal carcingenesis with no apparent changes in the inflammatory response [17], [32], [33].

The anti-inflammatory activity exerted *in vivo* by 8-oxoGTP or 7,8-dihydro-8-oxo-deoxyguanosine (but not by 8-oxoG) indicates that some oxidation by-products can modulate immune functions [34], [35]. The mechanism by which Mutyh influences the inflammatory response remains however to be ascertained. It is possible that repair intermediates (apurinic sites and/or single strand breaks) generated by the removal of A incorporated opposite DNA 8-oxoG might act as a signal triggering/amplifying the cascade of inflammation processes. Supporting evidence of a major role of repair intermediates in mediating several biological processes is the block in cell proliferation and activation of cell death induced by accumulation of abasic DNA damage in APE/REF1-defective human cells [36]. Alternatively Mutyh might act as a fine tuner of the redox function of the APE/REF1 endonuclease. This enzyme displays a powerful redox activity [37] that regulates the expression of multiple transcription factors involved in the regulation of the immune and pro-inflammatory responses [38], [39]. Finally Mutyh might play a role in cellular functions needed for an efficient native and adaptive immunity and its inactivation might result in an impairment of correct cellular maturation and ability to respond to inflammatory stimuli. These functions might not be necessarily mutually exclusive and could be the link between processing of DNA damage and inflammation processes.

In conclusion we show here that Mutyh activity is needed for eliciting the intense inflammatory response following an acute oxidative stress and is also involved in its attenuation during the chronic phase, probably by affecting the local occurrence of regulatory T cells. In view of the cancer prone phenotype associated with inactivation of the human *MUT*YH gene, it is tempting to speculate that the inability of *Mutyh* - defective mice to properly resolve the acute cellular response to external inflammatory stimuli and the consequent chronic persistence of the response might also plays a role in colorectal carcinogenesis in humans.

## Materials and Methods

### Animals

Animal care followed the directives of the EC Council (86/609/EEC). The experimental protocol was approved by the National Committee of the Italian Ministry of Health (authorization No. 162/2008-B). Mice were housed under pathogen-free conditions in the Laboratory Animal Services of Istituto Superiore di Sanità. *Mutyh^−/−^* mice were kindly provided by J.H. Miller [40]. Genotypes were confirmed by polymerase chain reaction of tail DNA using specific oligonucleotide primers [7]. The experimental protocol was approved by the Italian Ministry of Health. Acute colitis was induced in 10 week-old mice by treatment with 3%DSS (MW: ∼36,000–50,000, ICN Pharmaceuticals, Costa Mesa, CA, USA) in drinking water for 1 week followed by 2 weeks of tap water (1 cycle). The control groups were given ordinary tap water during the whole period. Body weights were recorded daily. Chronic colitis was induced by 8 cycles of 1.5%DSS treatment. For histology and immunohistochemistry, cross-sections of colon were dipped in Tissue Freezing Medium (Electron Microscopy Sciences, Hatfield, PA, USA) and kept at −80°C or fixed in periodate-lysine-paraformaldehyde (PLP) [41]. For DNA and RNA isolation, tissue sections were minced and snap frozen in liquid nitrogen within a few minutes after euthanasia (cervical dislocation).

### Histology and immunohistochemistry

Cryostatic sections were stained with hematoxylin/eosin or processed for immunohistochemistry. Sections were incubated with anti-mouse antibodies against CD4 (Abcam, Cambridge UK), CD8 (Oxford Biotechnology, Cambridge, UK), CD45R/B220 (a subset of mouse CD45 isoforms predominantly expressed on B lymphocytes; BD Pharmingen, San Diego, CA, USA), CD11b (a marker of monocyte/macrophage lineage; BD Pharmingen), PAX-5 (a marker for B lineage-committed cells; BD Biosciences, San Jose, CA, USA), Foxp3 (e-Bioscience, San Diego CA, USA). To immunostain Germinal Centers (GC), the sections were incubated with biotinylated peanut agglutinin lectin (PNA), whose binding to a common carbohydrate sequence distinguishes monocytes, macrophages and particularly centroblasts (Vector Laboratories, Burlingame, CA, USA). In CD4/Foxp3 double immunostaining experiments, binding of the primary Ab (anti CD4) in the first label was detected using avidin-biotin alkaline phosphatase (Thermo Scientific, Rockford, IL, USA) and Ferangi Blu (Biocare, Concord, CA, USA) as chromogen. Before the second staining with anti-Foxp3, frozen tissues were subjected to Ag retrieval by heat treatment in citrate (pH 6) buffer. The second label was detected using an indirect avidin-biotin phosphatase (Thermo Scientific) and Vulcan fast Red (Biocare) as a chromogen.

### Nucleic Acids extraction

DNA and RNA were extracted from colon tissues using the Puregene DNA Purification Kit (Gentra Systems, MN, USA) and QIAGEN RNeasy Midi Kit (QIAGEN, Valencia, CA, USA) according to the manufacturer's protocol. Briefly, colon sections were placed in 1.5 ml tubes of QIAGEN RLT® buffer and homogenized using the QIAGEN homogenizer. Homogenates were further processed according to the standard protocol for animal tissues. To determine purity, concentration and total yield of RNA samples, absorbance was determined spectrophotometrically at 260 and 280 nm and verified by the Agilent Bioanalyzer (Santa Clara, CA, USA) according to manufacturer's instructions.

### 8-oxoG determinations

8-oxodG was measured by HPLC/EC as previously described [42]. Following DNA extraction, RNase treatment and enzymatic hydrolysis, DNA hydrolysate was analysed by HPLC/EC (Coulochem I, Esa, Chelmsford, MA, USA) using a C18 250×46 mm 5 µm Uptishere column (Interchim, San Pedro, CA, USA) equipped with a C18 guard column. The eluent was 50 mM ammonium acetate, pH 5.5, containing 9% methanol, at a flow rate of 0.7 ml/min. Deoxyguanosine was measured in the same run of corresponding 8-oxodG with a UV detector (model SPD-2A; Shimadzu, Kyoto, Japan) at 256 nm.

### Microsatellite instability

For analysis of microsatellite instability (MSI), genomic DNA, isolated from the colon mucosa and the tail of the same mice, was analysed at mononucleotide *L24372*, *Aa006036* and dinucleotide *D1Mit62* and *D6Mit59* microsatellites. Primers, labelled with the fluorescent compound FAM were previously described [43], [44]. PCR reactions were carried out in a reaction buffer containing DNA (100 ng), primers (2 pmol/µl), dNTPs (200 mM), 0,5 U/µlTaq polimerase (Applied Biosystems, Foster City, CA, USA). After denaturation (95°C for 2 min), DNA was amplified by 25 cycles (30 s at 95°C, 30 s at 55°C, 30 s at 72°C). Amplification products (10 µl) were digested with 0,4 U/µl T4 DNA polymerase (Roche Diagnostic, Indianapolis, IN, USA)(30 min, 37°C) denatured in deionized formamide (2 min, 95°C) and analyzed with an ABI Prism 310 automatic sequencer by the GeneScan software (Applied Biosystems).

### Gene Expression Analysis

Gene expression was analysed using low-density arrays. Briefly, total RNA was converted to single stranded cDNA by reverse transcriptase and High-Capacity cDNA Archive Kit (Applied Biosystems, Foster City, CA, USA). cDNA was used for determination of gene expression level using TaqMan Low Density Arrays (Applied Biosystems). Relative quantification values have been normalized to the hypoxanthine phosphoribosyltransferase 1 housekeeping gene and then normalized to a calibrator sample (untreated wild-type colon sample) according to the 2^−ΔΔCt^ comparative threshold cycle (C_t_) method. Amplification was performed with ABI-Prism-7900 Sequence Detection System (Applied Biosystems) and mRNA was quantified using the CT method of relative quantification using SDS software version 2.2.1 (Applied Biosystems).

### Computational analyses

To analyse differences between experimental groups at single-gene expression level, the STATA statistical package was used [45]. To verify the independence of samples, non-parametric two-sample Wilcoxon rank-sum (Mann-Whitney) test for unmatched data was adopted. Genes were ranked on the basis of the discriminative ability of their *P*-values and data are shown as fold-change ratios between different subgroups with box-whisker plots graphically showing variations in gene expression levels among experimental groups. To perform PCA, SAS/STAT (http://www.sas.com/technologies/analytics/statistics/stat/index.html) was used. The effect of genotype, treatment and the mutual interaction of these two variation sources on the component scores were assessed by Analysis of Variance (General Linear Model, SAS).

## Supporting Information

Figure S1 Unsupervised PCA analysis of changes in gene expression in wt and *Mutyh−/−* mice exposed to DSS.
(0.11 MB TIF)
