# Supplementary figures and images for: The Mutyh Base Excision Repair Gene Influences the Inflammatory Response in a Mouse Model of Ulcerative Colitis
Source: PLoS One. 2010 Aug 10;5(8):e12070. doi: 10.1371/journal.pone.0012070 (PMC2919403; doi:10.1371/journal.pone.0012070)

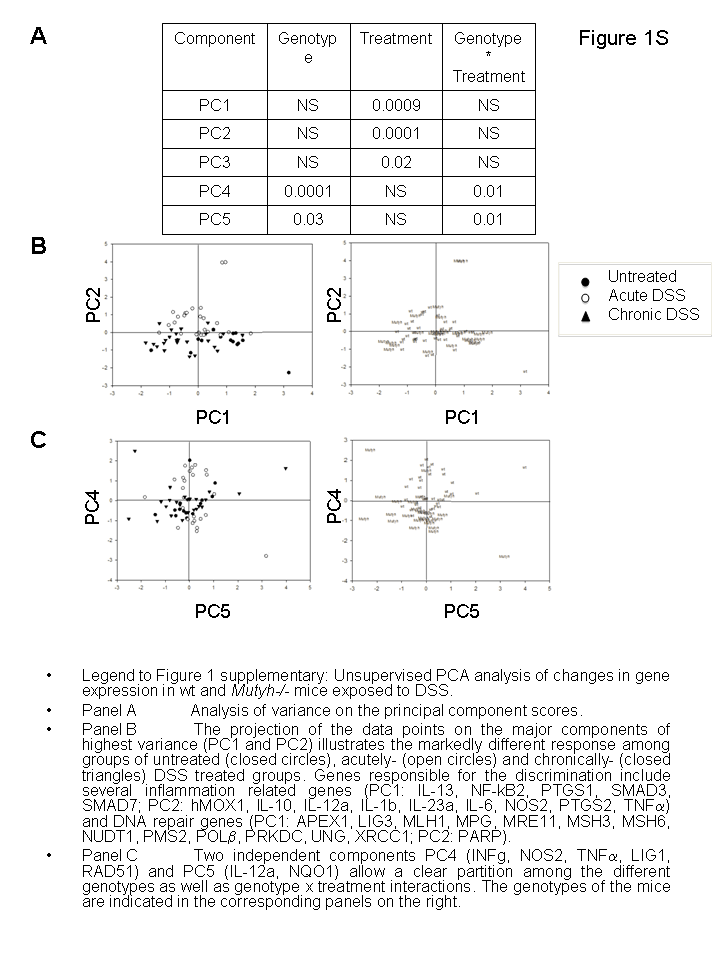

Supplement: Figure S1 — Unsupervised PCA analysis of changes in gene expression in wt and Mutyh−/− mice exposed to DSS. (0.11 MB TIF) [file pone.0012070.s001.tif]
